# Retraction Note: Integrative analysis of the circRNA–miRNA regulatory network in atrial fibrillation

**DOI:** 10.1038/s41598-021-96813-7

**Published:** 2021-08-23

**Authors:** Zhong‑bao Ruan, Fei Wang, Qiu‑ping Yu, Ge‑cai Chen, Li Zhu

**Affiliations:** Department of Cardiology, Jiangsu Taizhou People’s Hospital, Taizhou, 225300 People’s Republic of China

Retraction of: *Scientific Reports* 10.1038/s41598-020-77485-1, published online 24 November 2020

The Editors have retracted this article.

After publication, concerns were raised with respect to overlap with another publication by the authors^1^. Post-publication peer review confirmed that Figure 1A and 1B in this article and Figure 2B and 2D in^1^, Figure 2 in this article and Figure 3 in^1^, and Figure 3 in this article and Figure 7 in^1^ are the same. Additionally, data shown in Tables 3 and 4 in this study and Tables 2 and 3 in^1^ are also identical. This article is therefore redundant.

All Authors disagree with the retraction.
